# Deformation Behavior of Foam Laser Targets Fabricated by Two-Photon Polymerization

**DOI:** 10.3390/nano8070498

**Published:** 2018-07-06

**Authors:** Ying Liu, John H. Campbell, Ori Stein, Lijia Jiang, Jared Hund, Yongfeng Lu

**Affiliations:** 1 Department of Electrical and Computer Engineering, University of Nebraska-Lincoln, Lincoln, NE 68588-0511, USA; liuying900120@gmail.com (Y.L.); li.jia.jiang1985@gmail.com (L.J.); 2 Material Science Solutions, 2136 Westbrook Lane, Livermore, CA 94550, USA; campbelljh@comcast.net; 3 Schafer Livermore Lab, 303 Lindbergh Avenue, Livermore, CA 94551, USA; ostein@belcan.com (O.S.); jhund@belcan.com (J.H.)

**Keywords:** two-photon polymerization, low-density foam structures, laser targets, structure deformation, acrylate resin, Raman microspectroscopy

## Abstract

Two-photon polymerization (2PP), which is a three-dimensional micro/nano-scale additive manufacturing process, is used to fabricate component for small custom experimental packages (“targets”) to support laser-driven, high-energy-density physics research. Of particular interest is the use of 2PP to deterministically print millimeter-scale, low-density, and low atomic number (CHO) polymer matrices (“foams”). Deformation during development and drying of the foam structures remains a challenge when using certain commercial acrylic photo-resins. Acrylic resins were chosen in order to meet the low atomic number requirement for the foam; that requirement precludes the use of low-shrinkage organic/inorganic hybrid resins. Here, we compare the use of acrylic resins IP-S and IP-Dip. Infrared and Raman spectroscopy are used to quantify the extent of the polymerization during 2PP vs. UV curing. The mechanical strength of beam and foam structures is examined, particularly the degree of deformation that occurs during the development and drying processes. The magnitude of the shrinkage is quantified, and finite element analysis is used in order to simulate the resulting deformation. Capillary drying forces during development are shown to be small and are likely below the elastic limit of the foam log-pile structures. In contrast, the substantial shrinkage in IP-Dip (~5–10%) causes large shear stresses and associated plastic deformation, particularly near constrained boundaries and locations with sharp density transitions. Use of IP-S with an improved writing procedure results in a marked reduction in deformation with a minor loss of resolution.

## 1. Introduction

Two-photon polymerization (2PP) is a direct-write technology that has recently been used to create millimeter-scale laser target components to support the Department of Energy’s (DOE) High Energy Density (HED) research programs [1,2,3,4]. In the first published work in this area, Bernat et al. [5] and Jiang et al. [6] report the use of 2PP to print simulated fill tubes and low-density foam-like structures, respectively. More recently, Jiang et al. [7,8], Stein et al. [9], and Oakdale et al. [10] discuss details of the design, fabrication, characterization, and assembly of low-density foam targets.

Details of the 2PP process and technology have been reviewed recently [11]. In brief, polymerization is initiated by the simultaneous absorption of two photons by a photoinitiator in a reactive monomer/oligomer resin, and it thus depends on the square of the laser irradiance. In practice, an initiator is selected that has negligible absorption at the incident fundamental laser frequency but measurable two-photon absorption at the second harmonic. Because two-photon absorption cross-sections are very low, the probability of reaction initiation is negligible except near the laser focus. Therefore, photopolymerization only occurs at the peak of the focal irradiance and generates a volumetric polymer dot (“voxel”) that is generally smaller than the diffraction-limited spot size.

**Figure 1:**
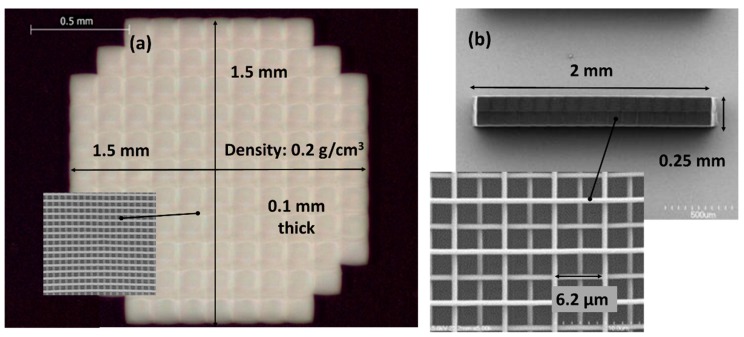
Examples of (**a**) foam plate and (**b**) a rod of two-photon polymerization (2PP) fabricated log-pile structures for laser target applications. The foam plate: 1.5 × 1.5 × 0.10 mm^3^ with a 4 × 4 × 2 μm^3^ beam lattice structure (density ~0.2 g/cm^3^). The foam rod: 2.0 × 0.25 × 0.35 mm^3^ with a 6.2 × 6.2 × 1.0 μm^3^ beam lattice structure (density ~0.1 g/cm^3^).

Typically, voxels range from 200–400 nm for fabricated structures that are similar to the ones reported here [6,7,8]. Structures are created by moving the repetition (rep)-rated laser beam (kHz to MHz) through the resin, thus generating overlapping voxels that, with proper scanning control, are built into the computer-aided design (CAD) three-dimensional (3D) shapes. Any unreacted resin is later removed during a post-writing development process, leaving behind a polymeric replica of the CAD design. Figure 1 shows examples of 2PP log-pile foam structures generated in our laboratory for laser target fabrication applications.

Structures described in this paper were made using one of two commercial resins: IP-Dip or IP-S. The IP-series are a family of proprietary acrylic resins plus initiator marketed by Nanoscribe GmbH for use with their commercial 2PP writing system. Using commercial resins is appealing, as it eliminates the need for custom formulation. In addition, acrylics are acceptable target materials as the atomic composition is largely carbon and hydrogen with minor amounts of oxygen. Many HED physics experiments, using foam or other polymer structures, require materials comprised of low atomic number elements because of the well-known variation in X-ray absorption (opacity) with atomic numbers [12]. Thus, the use of other common resins, such as hybrid organic/inorganic resins, for example, Ormocers, SZ2080 [13] or thiol-based resins [14], is not acceptable. Acrylic based low-density components and targets produced by 2PP are now being shot at major HED national research centers in the USA, including Lawrence Livermore National Laboratory (LLNL), Laboratory for Laser Energetics (LLE), Naval Research Lab (NRL), etc.

Acrylic resins, like IP-S and IP-Dip, are commonly used for 2PP fabrication of nano/microstructures. However, they are often limited by various materials and processing issues:

- inherent strength of the polymerized resin [14,15];
- strong sensitivity to writing conditions (peak irradiance and shots per site, i.e., dose) [16,17];
- low photo-conversion of resin to polymer [18,19,20];
- shrinkage during photopolymerization or development or both [6,9,20];
- stresses due to capillary forces during drying [6,9,21]; and,
- control of adhesion to the substrate [6,9].

Consequently, the user must consider each of these issues when selecting a given resin, the writing conditions, and the development method for a particular application. In this paper, we examine these issues by a combination of experiments and modeling, and suggest some possible methods for controlling the negative impacts of each in IP-Dip and IP-S resins. IP-Dip is particularly problematic. In contrast, IP-S shows significant improvement over IP-Dip, while still maintaining the required high CH content for target applications. The trade-off is in the line resolution, which is better in IP-Dip than in IP-S.

Shrinkage and deformation problems with the use of IP-Dip have been shown to stem largely from the low resin-to-polymer conversion (<50%) and an associated low modulus and yield strength of the polymer. Infrared and Raman spectroscopy are used to quantify the resin-to-polymer conversion by following the signature alkene vibrational bands. Finite element analysis (FEA) is used to simulate the degree of shrinkage and the resulting plastic strains that occur during the development and drying of 2PP log-pile foam structures.

## 2. Experimental

### 2.1. Photo-Resins and Properties

IP-Dip and IP-S commercial negative-tone, acrylate-based photoresists were used in this work (Nanoscribe GmbH, Eggenstein-Leopoldshafen, Germany), as they meet the low atomic number requirement for the proposed HED application. These resins are designed for use with Nanoscribe’s Dip-in Laser Lithography (DiLL) technology and they serve as both the immersion and photosensitive material. Specifically, the resins match the refractive index of the final focusing lens and achieve the highest numerical aperture (i.e., the best resolution) at a given magnification. 

IP-Dip has a low viscosity and is recommended for use in high-resolution applications requiring narrow line width. In contrast, IP-S is more viscous and designed for mesoscale printing at larger line widths. Elemental compositions and key properties of the resins are summarized in Table 1. The elemental compositions were determined using the procedure as reported in [6]. The resins are predominately CH_x_ with small amounts of O and traces of N thus satisfying the low atomic number requirement.

### 2.2. 2PP Microfabrication

Micro-fabrications were carried out using a Photonic Professional GT system (Nanoscribe GmbH [19]). Two-photon excitation was accomplished using the 780 nm frequency-doubled output from an Er-fiber laser (1580 nm, TEM_00_, M^2^ < 1.2, Toptica Photonics AG, Germany) operating at 80 MHz with a temporal pulse length ~100 fs. An integrated set of beam transport optics directed the laser output with circular polarization to a final focusing objective that dipped directly into the photoresist.

In the case of the IP-Dip resin, a 63X objective with a 1.4 numerical aperture was used for printing; whereas, with IP-S, the objective was 25X with a numerical aperture of 0.8. Table 2 provides a summary of the 2PP writing conditions used to fabricate the structures reported here.

The average incident laser power was measured by a photodiode that was located at the input to the focusing objective. The passive losses in beam propagation to the sample plane were assumed to be constant and accounted for in Nanoscribe’s as-built system calibration. The laser output power was controlled by an acousto-optic modulator that can be adjusted over a range of approximately 0 to 50 mW (average power). The beam diameter at focus, *D_b_*, was calculated by:

(1) $$D_{b}=\frac{2n\lambda }{\pi NA}\;,$$

where NA is the numerical aperture, *n* is the refractive index and *λ* is the wavelength.

Structures were created using the Nanoscribe built-in software package, DESCRIBE™ 2.5 (Nanoscribe GmbH, Germany), which generates General Writing Language (GWL) files directly. The Photonic Professional GT system (Nanoscribe GmbH, Germany) uses both a piezo stage and two coupled galvanic mirrors to write the structure. The galvanic mirrors allow for rapid x-y scanning at up to 10–20 mm/s over an area 200 µm in diameter when using the 63X final focusing objective or 400 µm in diameter when using the 25X objective lens. The vertical (z) motion is controlled by the piezo stage and the built-in z-drive of the focusing objective, which ranges up to several mm in height. The system can print structures with an area of up to 25 × 25 mm^2^ by using the motorized stage and “stitching” the structures together. The largest dimension of the structures that were fabricated in this application was 2 mm. The stitching accuracy is typically 1–4 microns [6]. 

The photoresist was deposited as a drop on a 25 × 25 × 0.7 mm^3^ glass substrate that was mounted in an aluminum sample tray. The tray, with substrate and resist, was inserted into the Nanoscribe GT housing that is attached to a precision piezoelectric-driven stage. All of the operations were carried out under yellow room lighting to avoid polymerization by single-photon absorption.

### 2.3. Structure Development, Drying, and Characterization

After exposure, the sample substrate with IP resin was removed from the holder and was developed at room temperature for 1 h in 50 mL of propylene glycol monomethyl ether acetate (PGMEA, Sigma-Aldrich, St. Louis, MO, USA), followed by a 1 h soak in 25 mL of isopropyl alcohol (IPA, Sigma-Aldrich, St. Louis, MO, USA). If a release layer was used, then the substrate plus the structure were removed from the IPA and immersed in the Microchem-specified release agent (Remover PG™, MicroChem Corp., Newton, MA, USA). The IPA was removed for either air or supercritical drying. Supercritical drying was accomplished using carbon dioxide (CO_2_) and a commercial drying system (SPI-DRY^™^, SPI Supplies, Inc., West Chester, PA, USA). Completion of the IPA solvent exchange with CO_2_ was determined using gas chromatography. The exchange was terminated when the residual IPA attained a level of 0.03% in the monitored CO_2_ effluent.

The surface morphology was characterized by optical and scanning electron microscopy (SEM). SEM images were obtained using a Hitachi model S4700 (Hitachi, Ltd., Tokyo, Japan). To obtain high-quality images, the samples were vapor coated with ~5 nm of chromium or gold. The imaging voltage was kept low (<10 kV) to avoid damaging the structures.

Raman spectra were recorded using a Raman microscope (Renishaw, InVia^™^ H 18415, UK) operating at an excitation wavelength of 785 nm and was focused onto the sample through a 50X objective lens (NA 0.75). Raman scattering was collected using the same lens. The average laser power and accumulation time used to record the Raman spectra were 10 mW and 10 s, respectively. Fourier transform infrared (FTIR) spectra were recorded on resins and polymerized thin films between 400 and 4000 cm^−1^ using a FTIR spectrometer (Nicolet™ iS50, Thermo Fisher Scientific Co., Waltham, MA, USA) equipped with diamond attenuated total reflection (ATR). Polymerized thin films (6 µm) were prepared by spin coating the resin on fused silica substrates, and then curing by single-photon polymerization at 395 nm for 10 min at 12 mW/cm^2^.

### 2.4. Finite Element Analysis

Finite element analysis was used to simulate the shrinkage and deformation of the log-pile structures and foam rods via COMSOL Multiphysics^®^ 5.3 software (COMSOL, Inc., Burlington, MA, USA), assuming a linear elastic response. Mesh configurations were created using COMSOL’s built in “fine mesh” to ensure solution convergence and computational efficiency. Constrained (fixed) boundary conditions were chosen to simulate the adhesion of the polymerized resin to the substrate.

Input material properties are given in Table 1. The effective Young’s modulus for the open cell foam, *E_f_*, was estimated using the correlation, as reported by Ashby [23]:

(2) $$E_{f}=E_{s}{\left(\frac{\rho_{f}}{\rho_{s}}\right)}^{2},$$

where, *ρ_f_* is the density of foam; and, *ρ_s_* and *E_s_* are the density and Young’s modulus, respectively, for the polymerized resin. 

Shrinkage was simulated by an equivalent thermal contraction of the structure using a stepped temperature drop and a user-defined thermal expansion coefficient and heat capacity for the foam and top layer. In contrast, a standard solid mechanics treatment was used to model the deformation caused by capillary forces. Further details are given in Section 3.5.

## 3. Results and Discussion

### 3.1. Structural Resolution of ID-Dip and IP-S Resin

The feature size of a microstructure fabricated by 2PP is determined by the size of the voxels, which is related to the induced photon intensity and sequent chemical reactions. The absorption of photons depends on the square of the light intensity, and the use of ultrashort pulses can start intense nonlinear processes at relatively low average power [24]. Theoretical studies have been established by several groups to investigate the dependence of linewidth that is based on nonlinear absorption [25,26].

In experiment, measurements of 2PP line widths versus laser power have been reported for three Nanoscribe resins (IP-DIP, -L780, and -G780) [6]. The work was carried out using the same Nanoscribe Professional GT system used here and at scan rates of 10 and 20 mm/s. A simple engineering model was used to predict the line characteristics vs. laser power and scan rate. Here, similar measurements and treatment are reported for IP-S.

**Figure 2:**
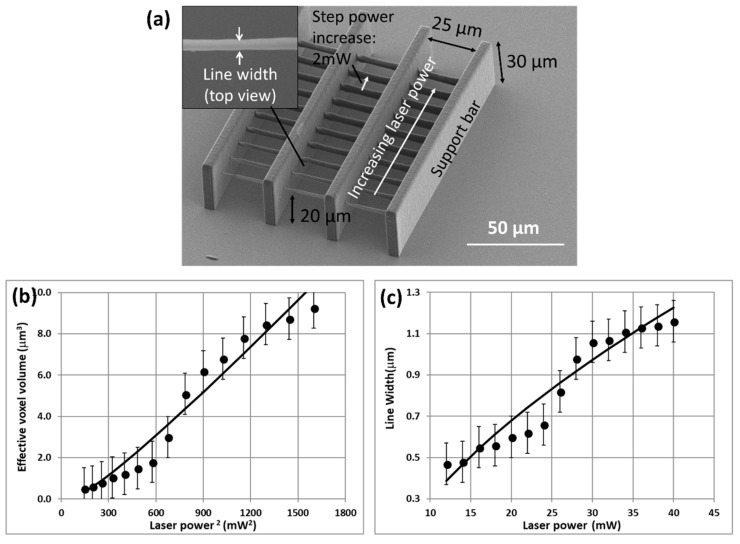
(**a**) Suspended line structures used to quantify 2PP line width vs. laser power for IP-S. Line widths were measured by scanning electron microscopy (SEM) (at normal incidence; see inset image) for lines printed in 2.0 mW stepped-increments of laser power; (**b**) Measured and calculated effective voxel volume vs. laser power^2^ and (**c**) linewidth vs. laser power for IP-S resin. The lines were calculated using the model described in Equations (3)–(6).

A set of support bars was first printed followed by a series of suspended lines normal to the bars (Figure 2a). The lines were printed using different laser powers and suspended to avoid complications due to interactions at the resin-to-glass interface. Each laser pulse above the threshold power initiated some degree of polymerization in an ellipsoidal-shaped voxel at laser focus. The fast laser repetition rate generated a continuous line of polymer comprised of closely overlapping voxels, each having an effective volume, *V_vox_*. The typical spacing between successive shots was ~0.1 nm at a scan rate of 10 mm/s and a laser repetition rate of 80 MHz, so the volume within a typical effective voxel received ~10^3^–10^4^ laser shots (Table 2).

The results are plotted in Figure 2b,c in terms of the effective voxel volume and linewidth as a function of the average laser power. The data were analyzed using a simplified engineering treatment that was initially suggested by Leatherdale and DeVoe [27], and more recently used by Thiel et al. [28]. These authors relate the absorbed dose in an initiated voxel volume, *V_i_* (nm^3^), to the laser operating conditions:

(3) $$V_{i}\sim k\;{\left(P_{a}-P_{t}\right)}^{2}\;t_{exp},$$

where *P_a_* is the laser average power (mW), *P_t_* is the threshold power (mW), *t_exp_* is the exposure time (s), and *k* is a proportional constant. The square dependence on power is due to the two-photon nature of the process and, thus directly proportional to the square of the per-pulse peak laser irradiance above the threshold. Note that we report the results in terms of the system average output power, rather than peak irradiance to simplify comparison with the typical system operational parameters. The average laser power was monitored and controlled during system operation.

Making use of the fact that the exposure time was inversely proportional to the scan rate (*R_s_*) and assuming the effective polymerized voxel volume, *V_vox_* (nm^3^), was proportional to the initiated volume, *V_i_*, led to:

(4) $$V_{vox}\sim k^{\prime }{\left(P_{a}-P_{t}\right)}^{2}/R_{s},$$

Experiments showed the polymerized voxel was ellipsoidal with diameter, *D,* and length, *Z*, perpendicular and parallel to the beam propagation direction, respectively, giving a geometric volume:

(5) $$V_{vox}=\frac{\pi D^{2}Z}{3}=\;\frac{\pi D^{3}A_{r}}{3},$$

where *A_r_* is the line aspect ratio, *Z/D*, which for IP-Dip and IP-S was measured at 2.5 and 5.4, respectively. Note that the ratio of the aspect ratios for IP-S/IP-Dip is 2.2, in good agreement with the value of 1.8 for the ratio of the numerical apertures that were used for printing in IP-S (NA = 1.4) and IP-Dip (NA = 0.8).

Combining Equations (4) and (5) and recognizing that the linewidth (*L_w_*) equals the effective voxel diameter (*D*) under constant scan rate conditions leads to the useful correlation for linewidth vs. operating laser power:

(6) $$L_{w}=\;{\left[k^{\prime }\frac{3{\left(P_{a}-P_{t}\right)}^{2}}{\pi A_{r}R_{s}}\right]}^{\frac{1}{3}},$$

The measured and calculated effective voxel volume and linewidth for IP-S are plotted vs. (*P_a_* − *P_t_*)^2^ and (*P_a_* − *P_t_*) in Figure 2b,c, respectively. In general, the agreement is reasonable given the indicated error in linewidth measurements. Similar reasonably good agreement has been reported in prior tests using IP-Dip and other resins [6].

A threshold power (*P_t_*) of 6 mW was assumed for IP-S, which is equivalent to the value for IP-Dip and other resins that were determined at very low scan rates (~0.1 mm/s) [20]. This threshold agreed with the lack of detectable polymerization (i.e., lines) below 12.5 mW at the much greater scan rates used here (10 mm/s). Clearly, some degree of polymerization (gelation) occurs at powers between 6–12 mW; but the dose is insufficient to generate a structure that is capable of surviving development.

In certain cases, the polymerization rate can be varied by a change of laser power and scan speed. Some authors have reported changes in polymerization propagation and termination rates due to temperature gradients formed around the focal point during 2PP [29,30]. However, in situ temperature measurements have not revealed a significant heating effect on the polymerization process when working at close-to-threshold conditions [31]. Therefore, the effect of localized thermal accumulation on 2PP fabricated structures is not included in this fitting model.

The primary benefit of the analysis reported here is as an engineering tool that, by interpolation, can reliably predict the line dimensions at different laser operating conditions in a given resin. The major limitation is that the analysis is largely an empirical treatment and it does not address the details of the excitation and complex polymerization chemistry of the process.

### 3.2. Plastic Strain in Simple Beam Structures Written in IP-Dip and IP-S Resins

Polymers characteristically have low elastic moduli and yield strengths but can accommodate significant plastic strain before ultimate failure [32]. These characteristics are an advantage in many 2PP applications. For example, many photo-resins undergo some shrinkage during polymer conversion, as evidenced by the greater density of the polymer vs. resin phase. Typically, polymer shrinkage is less than 2%. Consequently, polymers can generally accommodate small amounts of plastic strain without failure, thus leaving the desired structure fully intact.

**Figure 3:**
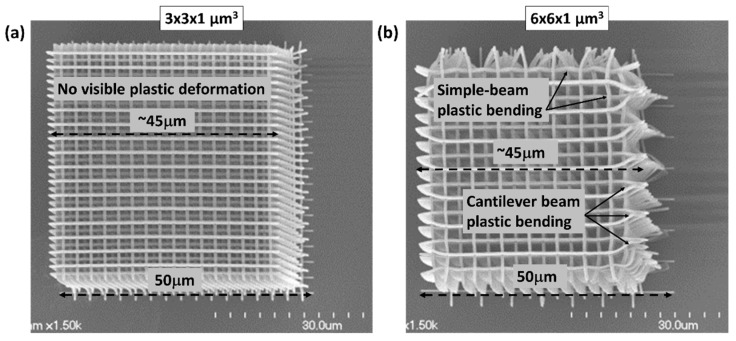
Log-pile structures with (**a**) 3 × 3 × 1 μm^3^ and (**b**) 6 × 6 × 1 μm^3^ cell size fabricated in IP-Dip resin with ~300–400 nm line width. Note the lack of observable plastic deformation at the smaller cell size in (**a**) in contrast to the visible bending in the simply-supported and cantilever beam sub-elements at the larger cell size in (**b**).

Problems tend to arise in 2PP fabrication when there are large strains, particularly in the cases of significant shrinkage or differential shrinkage during development. Examples of this are shown in Figure 3 for two log-pile like foam blocks fabricated in IP-Dip resin. The blocks were designed to be 50 × 50 × 50 µm^3^; yet, after drying, both had shrunk by ~10% to ~45 µm in width. The extent of the shrinkage was clearly visible in the rows at the base of the block where the fabricated lines contacted and adhered to the substrate. This degree of shrinkage is consistent with the shrinkage measured in other foam-like log-pile structures that were fabricated here and reported elsewhere [6,9,11,14].

Both structures in Figure 3 accommodated the shrinkage without evidence of plastic strain in the central portion of the structure. In such cases, one could attempt to compensate for shrinkage by simply designing and fabricating a proportionally larger structure.

Problems due to shrinkage were most evident at the boundaries of IP-Dip log-pile structures (Figure 3b). Large shear stresses developed at the fixed boundary between the polymer and the substrate. Also, certain structural elements, such as cantilever-type beams or simple beams that span long unsupported distances, exhibited large plastic deformation. Such deformations are visible in the structure in Figure 3b, but are noticeably absent for the shorter spans in Figure 3a.

**Figure 4:**
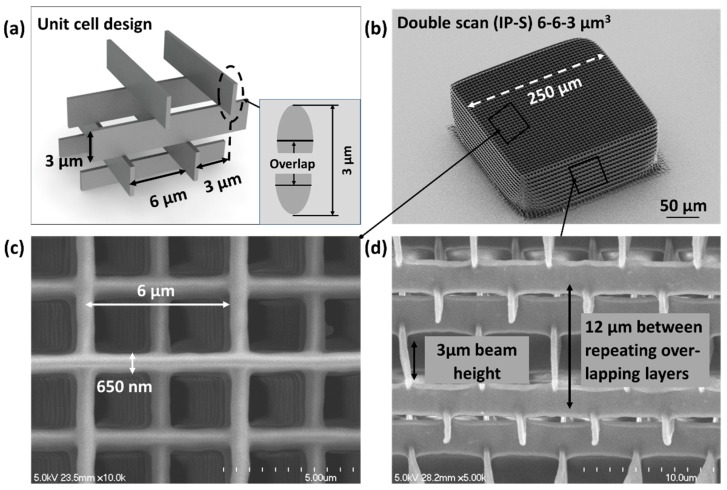
(**a**) The design of a log-pile structure with a 6 × 6 × 3 μm^3^ cell size fabricated using 50% overlapping double scans as described in the text. SEM images of (**b**) the 250 × 250 × 100 μm^3^ foam block fabricated in IP-S resin and in magnified views from (**c**) the top showing the linewidth and horizontal lattice spacing and (**d**) the side indicating the repeating overlap of every fourth layer, i.e., 4 × 3 um = 12 um.

Similar log-pile structures were written in IP-S (Figure 4). The linewidth and height were greater than those in IP-Dip because of the larger numerical aperture, as discussed in Section 3.1. Consequently, IP-S structures had to be fabricated using a bigger cell size (6 × 6 × 3 μm^3^, Figure 4a) to achieve foam densities that are equivalent to IP-Dip. The beams were laterally offset in successive layers with repeating alignment on every fourth vertical layer (Figure 4a). Each beam in the log-pile was fabricated using vertically offset and partially overlapping (50%) double scans to achieve the 3 μm height (Figure 4a inset). In general, the IP-S log-pile structures exhibited significantly less deformation than the similar structures that were written in IP-Dip (Figure 4b–d).

**Figure 5:**
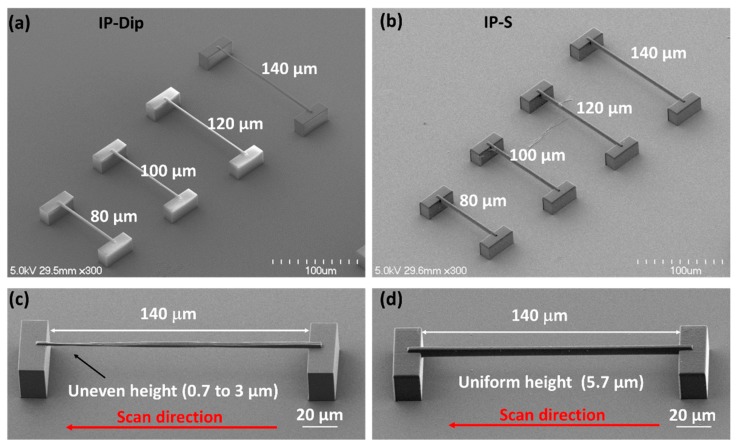
Simply supported beam structures fabricated in (**a**,**c**) IP-Dip and (**b**,**d**) IP-S resin. The scan direction was from right to left, as indicated by the arrow. The average laser power was 15 mW, and the scanning speed was 10 mm/s; see the text for further details.

**Figure 6:**
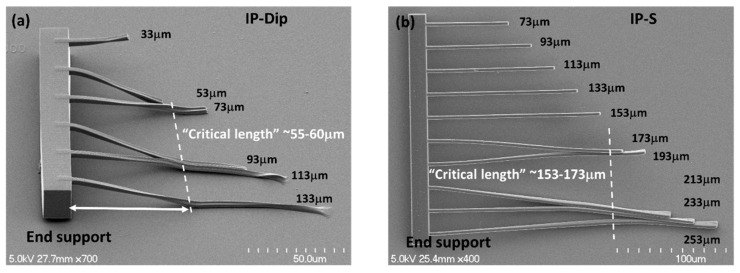
Cantilever beam structures of varying lengths with an integrated end support printed in (**a**) IP-Dip and (**b**) IP-S resin. The printed beam width is 3 µm with a lateral spacing between beams of ~20 µm and vertically suspended above the base substrate by ~20 µm. The “critical length” for collapse under capillary drying forces is indicated by the dashed line.

To better compare the strengths of structures written in IP-Dip vs. IP-S, we fabricated, developed, and air dried a series of simply supported and cantilever beams in the two resins (Figure 5 and Figure 6). Except for the beam length, all of the structures were designed and fabricated in the same way. The beam cross-sections were designed to be 3 × 3 µm^2^ and were fabricated using a 10-wide × 6-high scan grid, specifically 10 lateral scans at 0.3 µm line spacing and six vertical scans at 0.5 µm layer spacing. The average laser power was 15 mW, and the scanning speed was 10 mm/s. The beam structures were developed, rinsed, and air dried, as described in Section 2.3.

The final objectives used for IP-S and IP-Dip were 25X (NA = 0.8) and 63X (NA = 1.4), respectively (Table 2), with associated fabricated linewidths of ~0.4 and ~0.65 μm. Thus, adjacent scan lines overlapped more in IP-S than in IP-Dip. 

Simply supported beam structures that were fabricated in both IP-S and Dip showed no measurable plastic deformation after development and air drying (Figure 5). The only difference in performance between the two resins was (a) unevenness in the vertical thickness of the longest beam fabricated in IP-Dip (140 μm) and (b) greater overall vertical beam thickness as achieved in IP-S. The latter effect was due to the greater depth of field (Rayleigh range).

Figure 6 shows cantilever beams that were fabricated in IP-S and IP-Dip and then developed, rinsed in IPA, and air dried. Some of the longer beams plastically deformed to such an extent that they were connected in pairs as well as to the substrate. This is not surprising as the liquid meniscus that drives the capillary forces would be expected to span the spaces between the beams, as well as connect the beams to the substrate.

The effect of capillary drying forces on deformation in microscale cantilever and simply supported beams has been rather extensively studied because of the common use of air drying for solvent removal in many microfabrication processes (for example, [33,34,35,36]). The extent of deformation is generally characterized by a “critical length”, which refers to the distance from the beam attachment at the end support to the point of beam adhesion to a neighboring beam, the substrate, or both. For example, the critical length that was observed for the cantilever beams that were fabricated in IP-Dip was ~55–60 μm, whereas for IP-S, the value was ~153–173 μm (Figure 6).

Liu et al. [33] and Mastrangelo and Hsu [35] both provide closed-form solutions for estimating the critical length based on the polymer properties, beam dimensions, and inter-beam spacing. Although their mathematical approaches differ, they arrive at the same relationship:

(7) $$L_{c}={\left[\frac{3Ew^{3}d^{2}}{8\gamma \cos\left(\theta \right)}\right]}^{1/4}$$

where *L_c_* is the critical length (μm), *E* Young’s modulus (GPa), *w* beam width (μm), *d* beam spacing (μm), *γ* solvent surface tension (22 dyne/cm, IPA), and *θ* the wetting angle. Here, we assumed the structure was fully wetted (*θ* ~ 0°). Using the reported Young’s modulus for IP-S of 4.6 GPa (Table 3) gives a critical length of 157 um, which agrees well with the measured value. Repeating the same calculation for IP-Dip is problematic as Young’s modulus depends strongly on the writing speed and laser power (i.e., energy dose, J/cm^3^). For example, Lemma et al. [16] report a linear increase of 0.35 GPa/mW in Young’s modulus from ~0.75 to 3.6 GPa over a range in average laser power from 5–13 mW. The writing speed was 100 μm/s. In the work reported here, the writing speed was 10,000 um/s at a laser power of 15 mW. Therefore, Young’s modulus was expected to be lower. Equation (7) was used to estimate a Young’s modulus of ~0.1 GPa based on the observed cantilever beam critical length of ~60 μm (Figure 6a).

Mastrangelo [35] also treats the case of capillary collapse for a simply supported beam (i.e., a beam clamped at both ends). Using his results, we predicted the critical span to be ~160 and 400 μm for IP-Dip and IP-S, respectively. This agreed with the lack of collapse that was observed for the beam structures in Figure 5.

Polarization of the laser beam has been reported to affect the intensity distribution and thermal gradients around the focal spot thus leading to different polymerization rates, which can, in certain cases, affect the feature size and introduce small changes (~20%) in some mechanical properties [30]. We believe that impact of polarization effect on mechanical properties is likely to be small for our application compared to other effects. For example, Young’s modulus was estimated to be ~0.1 GPa for the IP-Dip polymerized structures written here, while the fully polymerized IP-Dip photoresist has a Young’s modulus of 4.5 GPa (Table 1). Also, at the employed high writing speed (10,000 µm/s), anisotropy in heat flow would be a second order effect for enhancing the mechanical stability of the foam targets. Besides, the beam is circular polarized for our writing process. Other research work has shown that the circular polarization of incident light could ensure a more spherical voxel within the xy-plane [37]. This avoids polarization-dependent linewidth between separate log-pile layers where the scan directions are perpendicular to each other.

Yoshimoto et al. [34] offers a different approach for describing plastic yield in micro-cantilever beams, with the resulting expression for the critical length in terms of the yield strength:

(8) $$L_{c}=w{\left[\frac{\sigma_{y}d}{6\gamma \cos\left(\theta \right)}\right]}^{1/2}$$

where *δ_y_* is the yield strength (MPa) and the other variables are the same as given above. To our knowledge, the yield strength for IP-Dip and IP-S has not been reported, so Equation 7 and the results in Figure 6 provide a means to estimate these values, specifically, *δ_y_* ~ 3 MPa for IP-Dip and ~20 MPa for IP-S.

### 3.3. Fourier Transform Infrared and Micro-Raman Vibrational Spectroscopy of Resin Conversion

**Figure 7:**
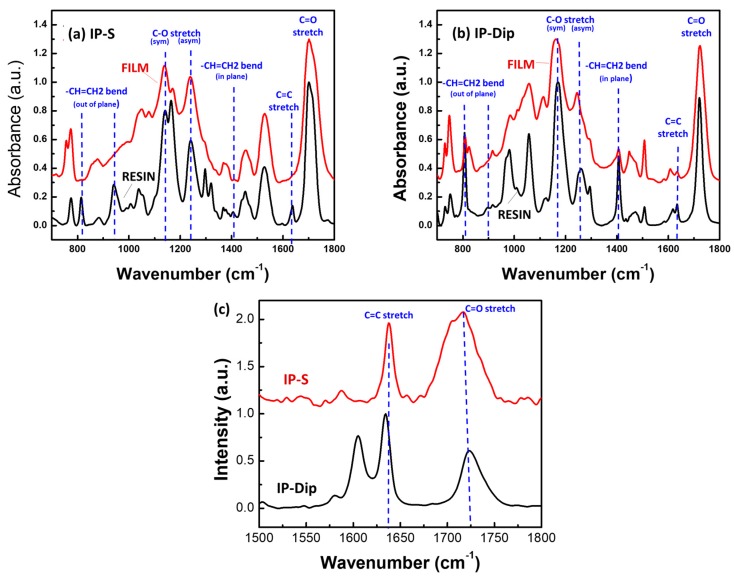
Fourier transform infrared (FTIR) spectra of the resin and fully cured film of (**a**) IP-S and (**b**) IP-Dip over the fingerprint region of 700–1800 cm^−1^. The bands associated with the terminal CH_2_=CH- stretching and bending modes are indicated on the spectra. (**c**) Raman spectra of IP-Dip and IP-S 2PP cured photoresists.

FTIR and micro-Raman vibrational spectroscopy were used to monitor the degree of polymerization in the IP-S and IP-Dip acrylic resins. Other recent studies have shown these techniques provide a wealth of molecular detail at the nano to microscale, about the extent of monomer/oligomer photo-conversion (see, for example, [11,14,20,38,39]). The characteristic vibrational bands that were associated with the CH_2_=CH-, C=O, and C-O groups that comprise the two resins are well known [40,41,42,43] and are clearly detected in both the FTIR and micro-Raman spectra (Figure 7).

FTIR bands are due to linear optical absorption by an oscillating dipole associated with the vibrations of a particular molecule or functional group [41,42]. In contrast, Raman bands are scattering phenomena and relate to the polarizability of the molecule or the molecular group. Specifically, Raman bands are associated with the radiation from an oscillating dipole that was induced by the incident laser electric field [40,41]. Thus, the two methods are complementary in that vibrational bands that are weak or not detected by one method may be detected by the other; this is often the case for our application.

In general, the FTIR spectra provide greater structural detail across the so-called molecular fingerprint region (~700 to 1800 cm^−1^), which includes characteristic stretching and bending modes of CH_2_=CH-, C=O, and C-O [42,43]. In addition, the method is insensitive to fluorescence from the initiator in the resin. The major drawback is that the FTIR spectrometer can only probe macroscopic samples. Raman microspectroscopy, on the other hand, has the advantage of being able to probe small volumes (<0.5 µm dia.) and it has greater sensitivity to the CH_2_=CH- stretching vibration at ~1600–1640 cm^−1^ [44]. This bond has a low dipole moment and it gives only weak FTIR bands, while the Raman signal is strong due to the large polarizability of the C=C bond. The main drawback to micro-Raman for our application was the interference caused by fluorescence from the initiator in the resin. 

FTIR spectra of unreacted resins and UV-cured films of IP-S and IP-Dip are shown in Figure 7. Table 3 summarizes the measured strength of the C=C stretching band and the three bending modes at ~1635, 1405, ~810, and ~940 cm^−1^, respectively. The intensity was normalized to the C=O band intensity because that group concentration is expected to remain constant in a given sample. The degree of conversion (DC) was calculated by comparing C=C stretching or bending mode intestines to a reference C=O band before and after UV photopolymerization [15,20]:

(9) $$\mathrm{DC}=\left[1-\left(A_{C=C}/A_{C=O}\right)/\left(A_{C=C}^{\prime }/A_{C=O}^{\prime }\right)\right]\times 100,$$

where *A_C=C_*, *A_C=O_*, *A*′*_C=C_,* and *A*′*_C=O_* are the integrated intensity of corresponding peaks in the polymerized and the unpolymerized resins. The IP-S spectra showed the expected result of complete reaction of the terminal alkene group after UV exposure. In contrast, the UV-exposed IP-Dip sample still contained ~17–29% unreacted C=C based on the ratios of the bands at ~810, ~1405, and 1635 cm^−1^ before and after UV exposure.

We next carried out micro-Raman measurements of lines that were written by 2PP in both resins using the conditions that are summarized in Table 2. The samples were developed and then examined using Raman microscopy, as described in Section 2.3.

Figure 7c shows the Raman bands for the C=O and CH_2_=CH- stretching modes after 2PP exposure. Both of the resins showed significant amounts of unreacted CH_2_=CH-. IP-Dip in particular showed low conversion, which is consistent with the trend that was observed in the UV-exposure results in Figure 7b. The reason for the difference in vinyl conversion of IP-Dip vs IP-S resins is difficult to assess without detailed knowledge of the chemical structure of these proprietary resins. Nevertheless, the IR and Raman data coupled with the elemental resin composition do offer a few hints. We assume here that the vinyl conversion continues (propagates) until the well-known free radical termination by oxygen (O_2_) [45]. Note that, based on the IR spectra and elemental composition results, IP-Dip is a fully vinyl-acrylate resin, whereas IP-S does show the presence of some amine functionality (amine bands at ~3300–3500 cm^−1^). We suspect that IP-Dip conversion becomes sterically hindered early on. In other words, molecular rearrangement is too slow (diffusion limited) to permit access to the unreacted vinyl groups before the small, highly mobile O_2_ terminates the reaction. In contrast, the full UV conversion of IP-S suggests adequate mobility to achieve full reaction before termination. The incomplete conversion of IP-S under 2PP is possibly due to insufficient initiation. Of course, use of other common organic chemistry structural analysis tools, for example, C^13^ and H^1^ nuclear magnetic resonance (NMR), gas chromatography-mass spectrometry (GC-MS), size exclusion chromatography (SEC), etc., could fully elucidate the structures of both resins. We have chosen not to do that here. Hopefully, the resin vendor will soon publish the structure making such analyses unnecessary.

The results suggest that low conversion is a major factor contributing to the large shrinkage and low modulus and yield strength of the IP-Dip foam structures. This agrees with prior work by Jiang et al. [12,15], who also reported low conversion by 2PP in custom resins with a resulting loss in mechanical integrity.

It is also probable that the C=C conversion varies through the width of the line, being greatest at the point of peak irradiance at the center of the focal spot and lower near the gaussian beam edges. Thus, the effective thickness of the line would be less than the observed thickness. Because the mechanical strength varies as approximately the cube of the line thickness, and then small negative changes in the effective line thickness would significantly weaken the part.

### 3.4. Fabrication of Foam Rods in IP-Dip and IP-S Resins

**Figure 8:**
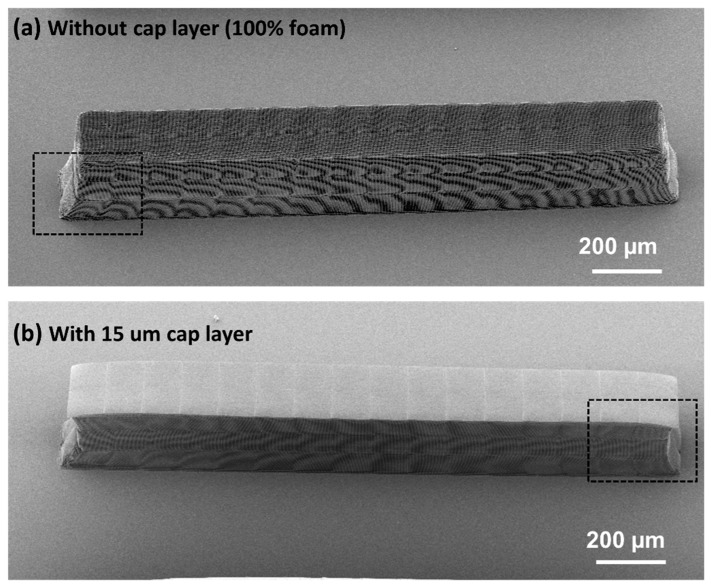
SEM images of 2 × 0.25 × 0.3 mm^3^ foam rod with x, y, z cell dimensions of 6.2 × 6.2 × 1 µm^3^ fabricated in IP-Dip (**a**) without and (**b**) with a 15-um-thick fully dense cap layer.

**Figure 9:**
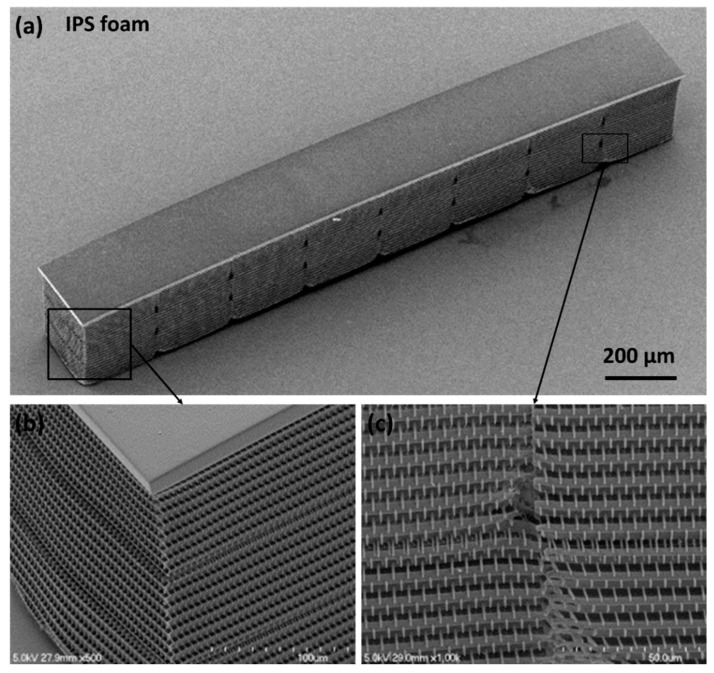
SEM images of (**a**) 2 × 0.25 × 0.3 mm^3^ foam rod with x, y, z cell dimensions of 6 × 6 × 3 µm^3^ and showing areas at higher magnification to illustrate (**b**) structure and (**c**) stitching boundary quality.

Figure 8 and Figure 9 show foam rods (2.0 × 0.25 × 0.35 mm^3^) fabricated in IP-Dip and IP-S, respectively, using the writing conditions in Table 2. Both foam structures have a design density of 0.10 g/cm^3^. One of the IP-Dip rods was fabricated as a 100% foam log-pile structure (Figure 8a), whereas the other had a 15-μm-thick cap layer that functioned as a laser ablator (Figure 8b). Only IP-S rods with the 15 μm cap layer were fabricated (Figure 9).

The IP-Dip and IP-S rods were comprised of a series of 125 × 125 × 100 μm^3^ and 250 × 250 × 100 μm^3^ sub-blocks, respectively. The ability to print larger sub-blocks for the IP-S rods is a direct result of the numerical aperture for printing (IP-S 0.8 vs. 1.4 for IP-Dip). The IPS sub-block width was designed to match the rod width, thereby reducing the number of stitching boundaries by eightfold over that for rods that were written in IP-Dip. The foam cell size for the IP-S rods was also designed to be larger (6 × 6 × 3 μm^3^) to offset the mass of the thicker beams and still achieve the 0.10 g/cm^3^ density goal. Each IP-S beam was fabricated with vertically offset and partially overlapping double scans (50%) to achieve a taller and stiffer (more reacted) structure (Figure 4a).

The IP-Dip rods showed the maximum deformation of ~6–7% at the interfaces of the foam with the substrate and with the solid cap layer (Figure 8). In contrast, the IP-S foam rods showed much less shrinkage and deformation in these interfaces (Figure 9). The largest defects/deformations in the IP-S rod occurred at the corners of the stitching boundaries (Figure 9b,c) and were consistent in size and location throughout the structure. Because these defects occurred at the outer surface of the rod, they did not impact the region of the target that was irradiated by the laser. Nevertheless, work is continuing on improving the process to eliminate these defects.

### 3.5. Analysis of Shrinkage and Deformation in IP-Dip Foam Rods

**Figure 10:**
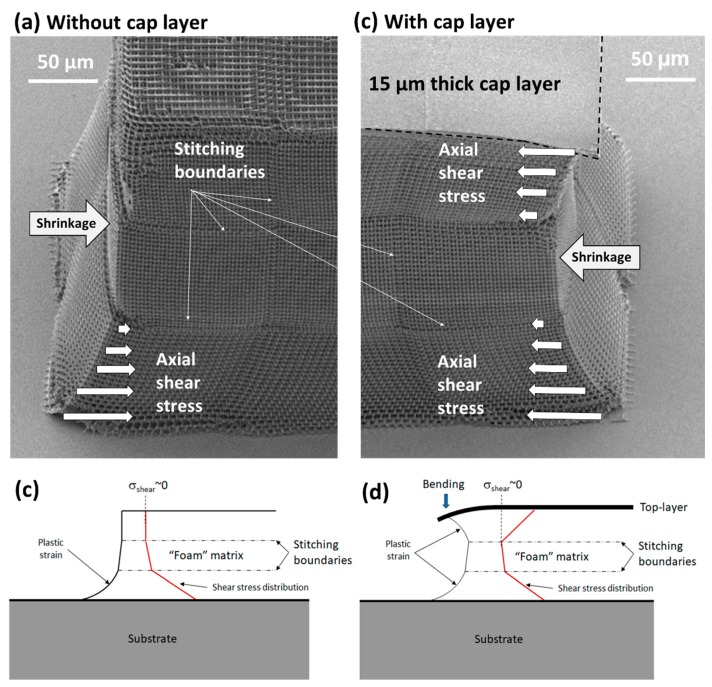
Details of the structural deformation of IP-Dip foam rods, specifically showing the regions within the dashed boxes in Figure 8. The SEM images show the ends of the rod (**a**) without and (**b**) with a 15-um-thick fully dense cap layer. Resin shrinkage during development and drying produced residual axial shear stresses and associated plastic strains in both the foam only and the foam with top cap, as shown schematically in (**c**,**d**) and also indicated in the SEM images; the arrow lengths are notional representations of the relative magnitude of the axial shear stresses.

Greater detail on the deformation of the IP-Dip rods at the interfaces between the foam-substrate boundary and the full-density cap layer are shown in Figure 10. Specifically, the images in Figure 10a,c provide magnified views of the rod end regions within the dashed boxes in Figure 8.

Comparison of the designed and measured dimensions of the IP-Dip foam rods indicated ~10% maximum axial shrinkage at the top of the foam rod. This is consistent with the measured shrinkage in other foam-like structures that were fabricated here and reported elsewhere [6,9]. The images in Figure 10 show that the axial shear stresses (and strains) due to shrinkage were greatest near “fixed” boundaries that were constrained by adhesion of foam to the glass substrate and to the full density top cap. Also, the stitching boundaries (Figure 10a,c) are regions of inherently lower strength that in some cases have been observed to cause layer-to-layer delamination under shrinkage-induced shear strains.

The axial shear stresses are indicated schematically in the SEM images in Figure 10a,b where the arrow lengths are notional representations of the relative magnitude of the stresses. Shear stresses in the lateral direction also exist, but are smaller.

**Figure 11:**
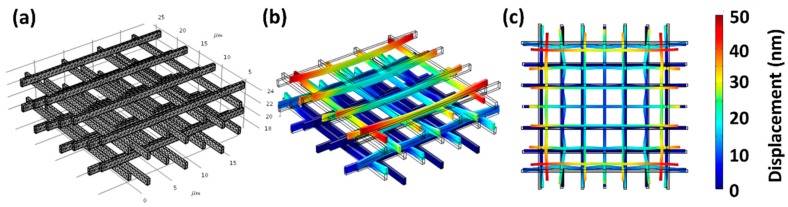
(**a**) Mesh configuration and (**b**,**c**) finite element analysis (FEA) simulation of shrinkage-induced deformation for 24 × 24 × 8 μm^3^ log-pile block fabricated in IP-Dip with 6.2 μm line spacing. See text for details.

**Figure 12:**
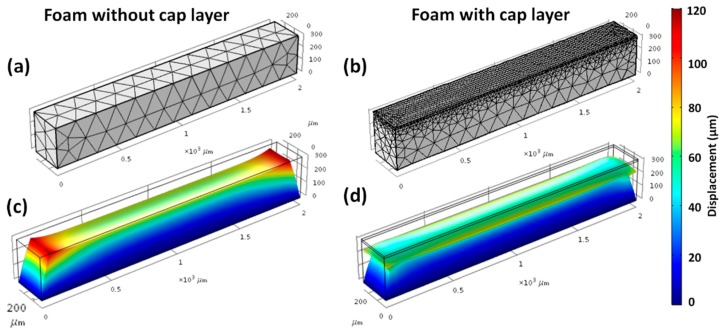
Mesh configuration used for the FEA simulations of foam rods fabricated in IP-Dip resin (**a**) without and (**b**) with a 15-μm-thick fully dense cap layer and (**c**,**d**) the computed deformation due to shrinkage.

Deformation of IP-Dip foam rods during the development process may be due to capillary forces and/or internal shrinkage due to the complete or partial removal of unconverted resin within the fabricated lines. Simulations were used to separately investigate the magnitude of these two effects by FEA. The geometry and the mesh configuration of a unit lattice and the foam rods with and without the cap layer are shown in Figure 11a and Figure 12a,b, respectively. The base of the lattice and the foam were constrained to simulate adhesion to an infinitely stiff substrate. In the case of the rod, the top cap was also assumed to fully adhere to the foam, although each can elastically respond to the applied stress.

We first considered the effects of meniscus drying forces. The capillary pressure, *P*, due to meniscus forces during air drying, can be estimated using the Young–Laplace equation:

(10) P~4γcos(θ)/L

where *L* is the effective pore diameter (or inter-beam distance), *γ* is the solvent surface tension, and *θ* is the wetting angle. Applying Equation (10) to the 6.2 × 6.2 × 1 μm^3^ foam unit cell and using a surface tension for IPA of *γ* = 22 dyne/cm and assuming a fully wetted structure (*θ* ~ 0°) gives an estimated maximum capillary pressure of ~8 kPa (~12 psi). The FEA of the foam rods predicted a maximum three-dimensional (3D) deformation of only ~130 nm over the entire length of the rod structure. This was less than half of the 2PP line width. Consequently, the deformation was expected to be well within the elastic limit with no permanent plastic strain, even at the very low estimated foam modulus value of ~0.1 MPa.

In contrast to the capillary drying effects, FEA simulations of the impact of shrinkage showed deformations that closely matched observations. Figure 11 shows show the FEA mesh configuration and the simulations results for a 24 × 24 × 8 μm^3^ beam lattice representing the microscale details of an individual building block of the foam rod. The lattice architecture had a horizontal beam (x, y) spacing of 6.2 μm, a beam height (z) of 1 μm, and a beam width (i.e., line width) of 0.4 μm. The simulation showed that shrinkage of the log-pile structures leads to uniform compression in the center region and plastic deformation at the end, as is consistent with the behavior shown in Figure 3. The vertical strain gradient (Figure 11b,c) was ~6–7 nm/micron over the 8 μm height and 24 × 24 μm base of this lattice structure. This predicted an expected total deformation of ~35 μm over the ~60 μm thick first layer of the 2 mm foam rod (Figure 10a,b).

The simulations of the magnitude of rod 3D deformation that is caused by shrinkage (Figure 12c,d) were also in reasonable agreement with the observed behavior in Figure 8 and Figure 10. The main differences between the simulations and observations were for the 100% foam rod. Specifically, the simulation showed a vertical expansion at the rod ends (Figure 12c), which is most likely due to the effect of the material Poisson ratio upon contraction. In the real rods, this did not occur, probably because of the shearing that was observed at the horizontal stitching boundaries, as shown in Figure 10a. In the case of the rod with a cap layer, the simulations compared most closely with the observations. These rods showed reduced shear at the stitching boundaries (Figure 10d) due to the axial constraint of the top layer. Moreover, the simulations correctly predicted the bending of the top cap at the ends of the rod where the shrinkage-induced stresses were the greatest. In this work, we assumed that the observed shrinkage was due to the effects of the unconverted resin, as discussed in Section 3.3. Other earlier studies of shrinkage of 2PP structures reached a similar conclusion [44,46]. However, a quantitative description of shrinkage at the molecular level remains elusive and is a subject of continued interest.

## 4. Summary and Conclusions

A series of low-atomic number (CHO) millimeter-scale foam laser targets with a 4–6 μm cell size were fabricated using 2PP. The targets are used to support HED physics research, thus driving the requirement that the foam contain only low-atomic number elements. The targets were comprised of a full-density 15 um cap layer at a 0.10 g/cm^3^ foam base. The cap layer served as an ablator.

Two commercial acrylic resins were evaluated for preparing the foam targets: (i) IP-Dip, a low viscosity resin designed for high-resolution printing with a large numerical aperture objective and (ii) IP-S a high viscosity resin for mesoscale printing using a lower numerical aperture. A fabricated linewidth in IP-S for different irradiance conditions was reported and compared to prior measurement on IP-Dip and also analyzed using a simple engineering model.

Infrared and Raman spectroscopy were used to measure the extent of 2PP polymerization by monitoring the C=C bond conversion. Although both IP-Dip and IP-S showed significant unconverted material, IP-Dip was worse; the results for IP-Dip agreed with observations that were reported by others. It was proposed that full or partial removal of unreacted resin from within the beams during development was the primary cause of the shrinkage and the resulting deformation observed in the final structures.

Simple end-supported beam test structures were fabricated in each resin to assess the polymer strength and the impact of capillary drying forces. The results showed that simple beams and foams fabricated with these resins were strong enough to support typical capillary drying forces of ~5–10 kPa (~0.7–1.5 psi) without plastic deformation. However, polymer linear shrinkage of up to 6–7% or more during resin development led to large structural plastic deformation in IP-Dip. Finite element analysis was used to simulate the effects of both capillary drying forces and the polymer shrinkage. Drying forces produced elastic deformations <0.5 μm, whereas shrinkage generated ~100× greater axial plastic deformation μm for these target structures.

A significant reduction in shrinkage-induced deformation and improvements in the structure strength and rigidity were achieved by using IP-S resin. Initial tests showed great improvements in the fabricated rod dimensional stability, with up to 4× fewer stitching boundaries and ~1/10th the shrinkage.

## Figures and Tables

**Table 1:** Composition and key properties of IP-Dip and IP-S resins. Unless otherwise noted, the physical and the mechanical properties are from Nanoscribe GmbH.

**Elemental Analysis of Resins**						
**Resin**	**Carbon (at.%)**	**Hydrogen (at.%)**	**Nitrogen (at.%)**	**Oxygen (at.%)**		**Empirical Formula**
IP-Dip	40.2	46	0.04	13.7		CH_2_N_0.001_O_0.34_
IP-S	31.5	54.1	5.8	11.8		CH_1.72_N_0.086_O_0.37_
**Physical and Mechanical Properties**						
**Resin**	**Density (liq) (g/cm^3^)**	**Density (s)** (g/cm^3^) *	**Young’s Modulus (GPa)**	**Hardness (MPa)**	Poisson’s Ratio ***	**Refractive Index**
IP-Dip	1.14–1.19	1.2	0.75–2.5 **, 4.5	152	0.35	1.52
IP-S	1.16–1.19	1.2	4.6	160	0.35	1.48

* [6], ** [16], *** [22].

**Table 2:** Top level summary of typical 2PP writing conditions used to prepare low-density structures reported in this work.

Parameter	Units	IP-DIP	IP-S
Final focusing power		63X	25X
Numerical aperture (NA)		1.4	0.8
Refractive index		1.52	1.48
Wavelength	µm	0.78	0.78
Beam waist (calculated)	µm	0.27	0.46
Focal spot area (calculated)	µm^2^	0.23	0.66
Pulse energy	nJ	0.19	0.21
Pulse length	fs	100	100
Pulse peak power	kW	1.9	2.1
Peak irradiance	kW/µm^2^	8.2	3.2
Pulse repetition rate	MHz	80	80
Average power	mW	15	17
Scan speed	µm/s	10,000	10,000
Line width (at 1cm/s scan)	µm	0.4	0.65
Shots/micron scanned		~8000	~8000

**Table 3:** Summary of FTIR peak intensities (normalized to the C=O peak) for CH_2_=CH- stretching and bending vibrational modes.

Band (cm^−1^)	Group and Mode	IP-S: Peak Intensity			IP-Dip: Peak Intensity		
		Resin	UV-Cured Film	DC	Resin	UV-Cured Film	DC
~1635	C=C stretch	0.06	0	100	0.07	0.02	71.43
~1405	C=C bend	0.03	0	100	0.34	0.08	76.47
~940	C=C bend	0.11	0	100	N.D.	N.D.	N.D.
~810	C=C bend	0.1	0	100	0.41	0.07	82.93
